# Pseudophakic malignant glaucoma - a case report

**Published:** 2019

**Authors:** Alin Ștefan Ștefănescu-Dima, Cornelia Andreea Tănasie, Maria Filoftea Mercuț, Irina Maria Mercuț, Mara Ionete, Carmen Luminița Mocanu

**Affiliations:** * Department of Ophthalmology, University of Medicine and Pharmacy of Craiova, Romania; ** Department of Physiology, University of Medicine and Pharmacy of Craiova, Romania; *** Department of Ophthalmology, County Clinical Emergency Hospital of Craiova, Romania

**Keywords:** malignant glaucoma, laser iridotomy, cycloplegics, pars plana anterior vitrectomy

## Abstract

**Purpose.** To report a case of malignant glaucoma in a pseudophakic female patient, with no history of glaucoma, resolved through pars plana anterior vitrectomy.

**Case presentation.** An 80-year-old female patient presented in our Emergency Department after a five-day history of pain in her left eye (LE). In the last day, the patient noticed marked visual loss and ocular pain. Visual acuity was light perception and Goldman tonometry was 80 mmHg in her LE. The biomicroscopy revealed absent peripheral and central anterior chamber (AC) and posterior chamber (PC) pseudophakia. Posterior segment ecography showed no vitreous or choroidal abnormalities. A peripheral laser YAG iridotomy was made and the patient was treated with intravenous 20% mannitol, topical timolol, topical brimonidine, and topical cycloplegics. 12 hours later, despite a patent iridotomy in the LE eye, intraocular pressure (IOP) was 55 mmHg, absent AC with severe corneal edema. The diagnosis of pseudophakic malignant glaucoma was made and laser YAG capsulotomy was performed with no resolution of symptoms and signs. 24 hours later, we performed pars plana anterior vitrectomy. Postoperatively, the AC depth increased and the IOP decreased to 20mmHg. After a week, the patient was discharged with hand movement perception visual acuity in her LE, 20 mmHg IOP, reduced corneal edema, normal depth AC. After a month, the corneal edema resolved, the visual acuity was 2/50, IOP was 20mmHg, and the AC had a normal depth.

**Conclusion.** Malignant glaucoma is a sight threatening condition, reported in pseudophakic eyes. Although, literature describes cases solved by cycloplegics and laser YAG capsulotomy, our patient needed pars plana anterior vitrectomy for the resolution of symptoms and signs.

## Introduction

Malignant glaucoma is an uncommon condition, first described in 1869 by Von Graefe [**1**]. Originally, malignant glaucoma is defined by a hightened intraocular pressure associated with a shallow or absent anterior chamber despite a patent iridotomy [**2**], and, in the absence of choroidal or vitreous effusions, unresponsiveness to miotics, with relief from cycloplegic therapy [**3**]. Typically, it is described as occurring after trabeculectomy in hypermetropic patients with angle closure glaucoma [**4**] and it is known as a condition with poor visual outcome [**1**], despite proper treatment.

Malignant glaucoma was first described after filtration surgery [**1**] but it can occur after cataract surgery from postoperative day one to many months [**5**], laser capsulotomy/ iridotomy [**6**,**7**], cyclophotocoagulation [**8**], penetrating keratoplasty [**9**] or spontaneous [**10**].

During time, some etiopathogenic theories were described, but the most accepted one suggests an anatomical connection between the lens, ciliary processes and anterior face of the vitreous, which causes the deviation of aqueous behind or into [**2**] the vitreous cavity, increasing the volume of the vitreous or detaching it from the retina and resulting in anterior movement of the diaphragm formed by the iris and the lens [**11**].

We described an uncommon case of malignant glaucoma, ten years after cataract surgery in a patient with no history of glaucoma.

## Case report

A 80-year-old female patient presented in our Emergency Department accusing pain in her left eye (LE) and decreased visual acuity. The symptoms appeared 5 days before and increased gradually. Visual acuity was light perception and Goldman tonometry was 80 mmHg in her LE. The biomicroscopy revealed severe conjunctival injection, absent central and peripheral anterior chamber (AC), iridocorneal touch centrally and posterior chamber (PC) pseudophakia. An ultrasound B-scan was performed to rule out posterior segment pathology, which showed no vitreous or choroidal abnormalities. The patient was operated for cataract 12 years before in the right eye (RE) and 10 years before in the LE, with no other ocular history.

In the RE, the visual acuity was 1, the Goldman tonometry was 17mmHg, the biomicroscopy revealed posterior chamber pseudophakia, otherwise normal biomicroscopy and normal fundus. 

A peripheral laser YAG iridotomy was performed immediately in the LE and the patient was treated intravenously with 20% mannitol, topical timolol, topical brimonidine, and topical cycloplegics. 12 hours later, intraocular pressure (IOP) in the LE was 55 mmHg, absent AC with corneal edema, despite the presence of a patent iridotomy. The diagnosis of malignant glaucoma was made and laser YAG capsulotomy was performed with no resolution of symptoms and signs. 

**Fig. 1:**
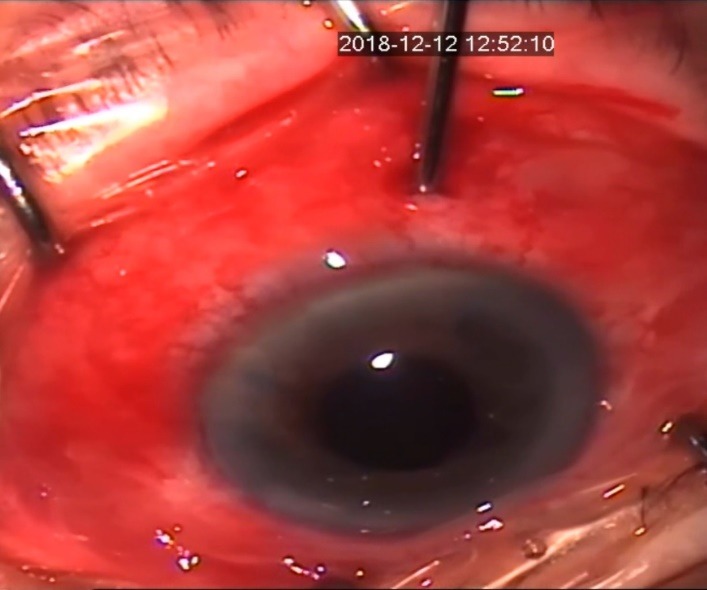
Pars plana anterior vitrectomy (intraoperative aspect)

**Fig. 2:**
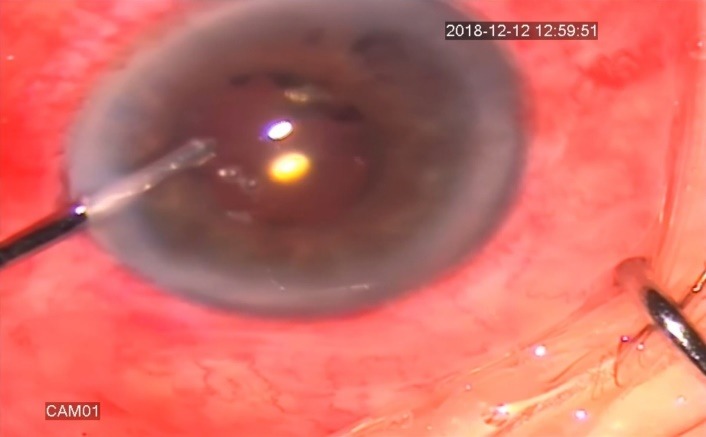
Aspiration of viscoelastic (intraoperative aspect)

24 hours later, we performed pars plana anterior vitrectomy with posterior capsulotomy and anterior hyaloidectomy, creating a direct connection between anterior chamber and vitreous cavity (**Fig. 1**,**2**).

**Fig. 3:**
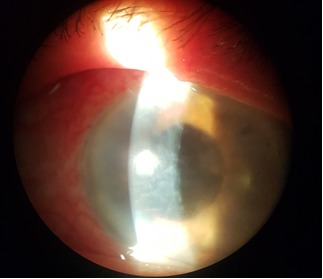
Biomicroscopic aspect - one week postoperatively

**Fig. 4:**
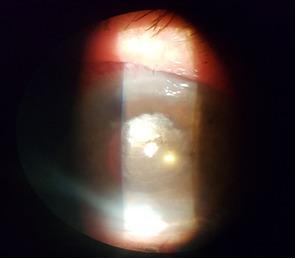
Biomicroscopic aspect - one week postoperatively

First day postoperatively, the AC depth increased and the IOP came down to 20mmHg. The patient was discharged after a week with hand movement perception visual acuity in her LE, 20 mmHg IOP, decreased corneal edema and normal depth AC (**Fig. 3**,**4**).

**Fig. 5:**
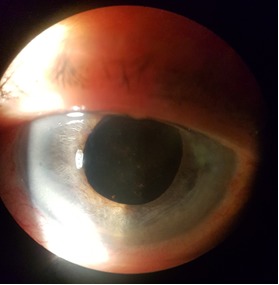
Biomicroscopic aspect of LE - first month postoperatively

**Fig. 6:**
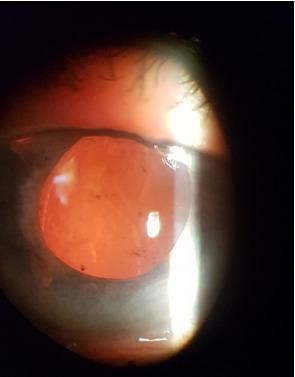
Biomicroscopic aspect of LE - first month postoperatively

**Fig. 7:**
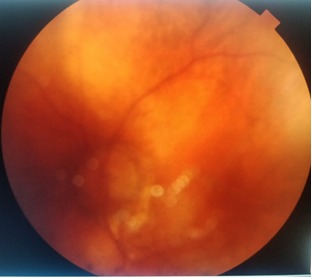
LE fundus - one month postoperatively

After a month, the corneal edema resolved, the visual acuity was 2/ 50, IOP was 20mmHg, and the AC had a normal depth. The fundus showed peripapillary atrophy, with normal contoured papilla, excavation C/ D 0.5 (**Fig. 5**-**7**).

**Fig. 8:**
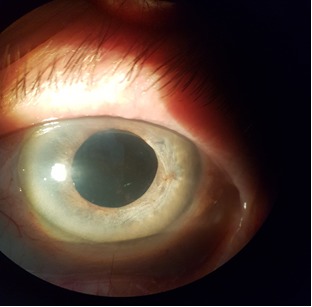
Biomicroscopic aspect of LE - two months postoperatively

Second month postoperatively, the visual acuity in the LE was 2/ 50, IOP 19 mmHg, and normal depth AC (**Fig. 8**). The patient is still under treatment with topical brimonidine and cycloplegics.

## Discussion

Malignant glaucoma, also known as cilio-lenticular block, ciliary block, or aqueous misdirection glaucoma [**2**] represents one of the most difficult types of secondary glaucoma to treat and manage. It is a very rare complication of all kind of ocular surgeries with an incidence of 2-4% [**12**]. 

It is characterized through increased IOP in the presence of a very shallow or flat AC, normal posterior segment, no response to miotics or peripheral iridectomy, and good response to cycloplegics [**2**]. 

The exact etiopathogenic mechanism is still uncertain but it is almost certainly associated with the anatomical relation between lens, anterior hyaloid, ciliary body and zonules [**13**]. Shaffer and Hoskins observed a posterior deviation of aqueous flow, which led to its accumulation behind a posterior hyaloid detachment and a forward displacement of the iris-lens diaphragm [**14**]. Chandler suggested that laxity of the zonules with vitreous pressure leads to anterior lens-iris movement [**15**]. Quigley proposed choroidal expansion to expand the vitreous pressure [**16**,**17**].

Pseudophakic malignant glaucoma is a rare complication which can appear after cataract surgery within various latencies, most frequent in the immediate postoperative period. The mechanisms through which malignant appears in pseudophakic eyes were studied during years. Chandler considered the cause is zonular weakness which lead to anterior subluxation of the IOL [**15**]. Showing that anterior chamber shallowing normalized after anterior hyaloidectomy [**18**], Tello C et al. proposed that an intact anterior hyaloid has an important role in the etiopathogeny of malignant glaucoma in pseudophakic eyes. In a study on 23 pseudophakic eyes with malignant glaucoma, Dave P et al. suggested zonular weakness secondary to pseudoexfoliation a possible cause of this pathology [**19**].

We report an unusual case of spontaneous aqueous misdirection that occurred with a significant delay from the cataract surgery, 10 years, in a patient with no history of pseudoexfoliation or glaucoma. Usually, this condition is reported in the immediate postoperative period, when inflammatory responses due to the surgical procedure are still active. In our knowledge, there is only one case described 16 years later after cataract surgery [**20**]. We consider that etiology is anterior displacement of the ciliary body and zonular weakness in the presence of an intact anterior hyaloid. 

Regarding the treatment, the initial recommended management is medical [**2**,**5**,**18**,**20**] and is reported to be successful in 50% of the cases [**21**]. In our case, the medical treatment with osmotic agents, cycloplegics and hypotonic associated with laser YAG iridotomy had no resolution of signs and symptoms. Also, YAG capsulotomy had no effect. Because 24 hours after medical treatment and YAG iridotomy and capsulotomy, the AC was still absent and the IOP up to 50 mmHg, we decided to perform pars plana anterior vitrectomy to create a direct communication between the vitreous cavity and AC. This involves anterior vitrectomy, anterior hyaloidectomy, and posterior capsulotomy. During the procedure, the AC deepened. The next days, the AC remained deep, the IOP decreased to 20 mmHg and the corneal edema decreased significantly. We observed the patient monthly, 6 months postoperatively and she remained stable, with 2/ 50 visual acuity in the LE, 20 mmHg, normal depth AC. 

## Conclusions

We reported an unusual case of malignant glaucoma that occurred in a pseudophakic patient with no history of glaucoma or pseudoexfoliation, with 10 years delay time from the cataract surgery, due to zonular weakness. Regarding the treatment, pars plana anterior vitrectomy with hyaloidectomy and capsulectomy proved to be a valuable option for our patient, with no relapse of sign and symptoms in a 6-month follow-up period.

**Financial Disclosures**

None of the authors has any financial or proprietary interests to disclose.
